# Antisepsis and Antiseptics

**Published:** 1896-02

**Authors:** B. F. Calhoun

**Affiliations:** Beaumont, Texas


For Texas Medical Journal.

### ANTISEPSIS AND ANTISEPTICS.

BY B. F. CALHOUN, M. D., BEAUMONT, TEXAS.

[Read at the Southeast Texas Medical Association.]

I HAVE been requested by you gentlemen to prepare an article on antisepsis and antiseptics. First, the primary elucidation of the principles of antisepsis originated with the surgical branch of the healing art, therefore it is but natural that we should look to this department of medical science for subsequent development and improvement. Have our expectations been realized, or has time, with ruthless hand, swept all record from the history of progress?

Let us investigate and see if our expectations have been realized. About twenty years ago, the principles of antisepsis first came into practical use in surgery, and from the very first period of its inception to the present time, has been one continued and uninterrupted progress. Within the past few years, the results of bacteriological research have completely revolutionized surgical pathology and surgical methods. A very large majority, if not all, of the acute and chronic inflammatory processes and lesions, as well as wound complications, which are brought to the attention of the surgeon, are due to micro-organisms. For this reason, it is necessary and expedient that the importance of bacteriology, as an integral portion of the science and art of modern surgery, be given due and proper recognition.

The very centers of life and functions are approached, with little or no fear of untoward consequences. This confidence is not the child of audacity, but the offspring of confidence and success. Abdominal surgery has sprung into bud and blossom beneath the kindly influence of modern aseptic and antiseptic methods. The thoracic cavity is invaded, and no ill result is anticipated. All of these triumphs were possible only upon one contingency—complete and thorough antisepsis.

But the greatest achievement of modern surgery is not so much in the triumph of either abdominal or cerebral surgery, nor, indeed, of any one or more particular class of operations. This lies in the method which has brought every organ within the domain and under the influence of the surgeon; it forms the very foundation upon which the superstructure rests. The victory and success of that method manifests itself no less in recovery from the simplest operation, than in that from laparotomy or craniotomy, or the extirpation of a cerebral tumor, without the formation of pus or the development of pyrexia. So great is the importance of this method, that the introduction of antisepsis marks the dawn of modern surgery.

Sir Joseph Lister, I believe, was the first to recognize and appreciate the fact that wound infection is dependent upon the presence and influence of micro-organisms. But he did much more than this, for he adopted measures of treatment, based upon this truth; he not only recognized the evil, and appreciated its source, but also formulated a systematic method of combatting these microscopic, yet omnipotent, as well as omnipresent, foes of the surgeon. His fame will rest safely and securely upon the establishment of these fundamental principles, even if every detail of the original method be supplanted by better ones. Indeed, this has taken place to some extent already, and will probably continue to do so; but the principles upon which they were founded remain absolutely unaltered and unshaken in every particular. The antiseptic and aseptic methods depend no less upon the researches of bacteriology than upon clinical experience, —indeed, in the development of these methods, both work together, hand in hand, the one announcing principles which the other puts into practice. The clinical and bacteriological proof that suppuration, pyemia, septicemia, erysipelas, and other similar complications, are due to the presence and influence of micro-organisms, is practically absolute. The germ theory of putrefaction is the foundation of the whole system of antiseptic surgery, and if this theory is a fact, it is a fact of facts that the antiseptic system means the exclusion of all putrefactive organism. This theory is founded upon a mass of correlated and substantial facts, experimental, clinical, physiological, pathological and bacteriological in character.

The statistics of all operations, major and minor, in the present period, as contrasted with those of the past, establish the fact that those septic diseases which were rife in private, but more especially hospital practice, have almost entirely disappeared. I can remember, during the late war, the great number of soldiers in the hospital, afflicted with gangrene, and a great many died in the hospital, from slight wounds, for the want of proper antiseptic treatment.

In 1867 and 1868, when I attended my first course of medical lectures, the professor of surgery instructed our class in the art of healing by producing suppuration, which would naturally prevent primary union; now modern surgery teaches us to secure primary union, and to prevent the occurrence of suppuration,—indeed, the formation of pus, at the present time, is looked upon as a tangible cause for reproach to the surgeon in charge. The rate of mortality consequent upon amputation and abdominal section has fallen nearly to zero, with the aid of antiseptic remedies. The history of the development and establishment of this great theory, which has so profoundly influenced the progress, not only of surgery, but the whole medical science as well, is the result of the labor, not only of one, but of hundreds of the world's choicest intellects; it constitutes one of the brightest of all the chapters in the record of human progress, and I am proud to say it does not belong to one nation exclusively, but is the common property of the civilized world.

Now, let us glance briefly at some of the benefits which the principles of antisepsis, which naturally include those of asepsis, have conferred upon humanity. We take the record of Lister, and that of Spence. Lister, with anticeptic precaution, and Spence, without antiseptic precaution, worked in the same hospital, upon the same class of patients, for practically the same condition. Spence lost, from infectious diseases, nearly five times as many patients as Lister. These results were not peculiar to the practice of Lister, the originator of the system. They have been corroborated and confirmed by every impartial observer and operator. Nussbaum states that in forty years experience in his clinics under himself, as well as his predecessors, deaths from wound diseases and wound complications were so common, prior to the introduction of antiseptic methods, that even those patients with the slightest injuries frequently succumbed to them. He furthermore states that, during the same period, erysipelas and abscesses were matters of daily occurrence, that eighty per cent of all wounds and sores were attacked by hospital gangrene, and nearly all cases of compound fracture terminated in death; but immediately upon the introduction of the antiseptic method, all of these diseases suddenly disappeared, and healing by first intention became the rule, instead of the exception. His statistics also show that, while for sixteen years previous hospital gangrene, erysipelas, pyemia and septicæmia, had never been absent from the wards of the Munich General Hospital, they suddenly vanished upon the introduction of antiseptic methods of wound treatment. Again, Dr. Thomas G. Moore, in one of his Pennsylvania Hospitals, reports as follows:

"From the spring of 1875 to the same period in 1879, a period of four years, there were performed 108 amputations, upon 100 patients. Of this number, 17 died; five of these deaths took place within the first thirty-six hours following the admission of the patient, and in each instance from recurring shock."

And he further states that he was greatly pleased to be able to report that during the past five years there had not occurred, in the surgical experience of the hospital, a single case of pyemia, and he believes that this result is due to the very perfect system of forced ventilation by the fan, and the scrupulous cleanliness of the wards and the free use of antiseptic remedies.

I could give reports of other eminent surgeons on the antiseptic method of wound treatment, but do not deem it necessary, for I believe over ninety per cent. of the surgeons of the present day recognize the great value of the modern method of antiseptic wound treatment. It is clearly the legal, as well as the moral, duty of every physician, who attempts the practice of surgery, to prevent the access of germs to the wounds of his patient, by every means within his power. It is not a mere matter of choice,—it is incumbent upon him; it is obligatory, or else he must expect to bear the stigma of merited reproach. The highest aim of the surgeon must be to guard the patient, both during and after the operation, against the injury and danger of wound infection; that is, in ideal work, where a wound is required to be healed quickly, it must be guarded against infection and the deposition and development of the agents of decomposition. Let me make this assertion right here, that with thorough and conscientious precaution, and the proper use of antiseptics, there is not, at the present time, any portion of the human body which is entirely without the pale of the dominion of the surgical sceptre, the scalpel.

It is hardly necessary, gentlemen, for me to attempt to give you the names of the large number of drugs possessing disinfecting and antiseptic powers—it would consume too much of your time. But, in closing this brief and somewhat imperfect résumé of the triumphs of antisepsis, I will say that the fear of suppuration, with its dreaded consequences, does not stay now the hand of the surgeon, as of old.

